# Identification of Transcription Factors Regulating CTNNAL1 Expression in Human Bronchial Epithelial Cells

**DOI:** 10.1371/journal.pone.0031158

**Published:** 2012-02-16

**Authors:** Yang Xiang, Xiao-Qun Qin, Hui-Jun Liu, Yu-Rong Tan, Chi Liu, Cai-Xia Liu

**Affiliations:** Xiangya School of Medicine, Central South University, Changsha, Hunan, China; Ottawa Hospital Research Institute, Canada

## Abstract

Adhesion molecules play important roles in airway hyperresponsiveness or airway inflammation. Our previous study indicated catenin alpha-like 1 (CTNNAL1), an alpha-catenin-related protein, was downregulated in asthma patients and animal model. In this study, we observed that the expression of CTNNAL1 was increased in lung tissue of the ozone-stressed Balb/c mice model and in acute ozone stressed human bronchial epithelial cells (HBEC). In order to identify the possible DNA-binding proteins regulating the transcription of CTNNAL1 gene in HBEC, we designed 8 oligo- nucleotide probes corresponding to various regions of the CTNNAL1 promoter in electrophoretic mobility shift assays (EMSA). We detected 5 putative transcription factors binding sites within CTNNAL1 promoter region that can recruit LEF-1, AP-2α and CREB respectively by EMSA and antibody supershift assay. Chromatin immunoprecipitation (ChIP) assay verified that AP-2 α and LEF-1 could be recruited to the CTNNAL1 promoter. Therefore we further analyzed the functions of putative AP-2 and LEF-1 sites within CTNNAL1 promoter by site-directed mutagenesis of those sites within pGL3/FR/luc. We observed a reduction in human CTNNAL1 promoter activity of mutants of both AP-2α and LEF-1 sites. Pre-treatment with ASOs targeting LEF-1and AP-2α yielded significant reduction of ozone-stress-induced CTNNAL1 expression. The activation of AP-2α and LEF-1, followed by CTNNAL1 expression, showed a correlation during a 16-hour time course. Our data suggest that a robust transcriptional CTNNAL1 up-regulation occurs during acute ozone-induced stress and is mediated at least in part by ozone-induced recruitments of LEF-1 and AP-2α to the human CTNNAL1 promoter.

## Introduction

Catenin alpha-like-1(CTNNAL1) was first characterized as a 2.45-kb transcript that was down-regulated in human pancreatic cancer cells [1]. With 734 amino acids, the predicted CTNNAL1 polypeptide has similarities to human vinculin and α-catenin, especially in the N-terminal region, which contains binding sites for β-catenin, talin and α-actinin [2]. Amphipathic helices in the C-terminal homology region corresponding to α-catenin contain potential binding sites for the tight junction protein ZO-1 and the actin cytoskeleton [2], [3], suggesting that CTNNAL1 may act as a cytoskeletal linker protein.

Recently, CTNNAL1 was identified as a part of the Rho signalling pathway, serving as a scaffold protein for Lbc [3], a member of the dbl family of Rho guanine nucleotide exchange factors (GEFs) [4], [5]. Rho GTPases play important roles during organization of the actin cytoskeleton and formation of focal adhesions [6]. Wiesner C et al [7] reported that CTNNAL1 also interacts with the IκB kinase (IKK)- β, a key component of the NF-κB signaling pathway. Ectopic expression of CTNNAL1 augmented NF-κB activity, promoted cell migration and increased cell resistance to apoptosis.

In the previous study, we found that CTNNAL1 mRNA decreased in the lung of OVA-sensitized asthma animal model. There was a negative correlation between the pulmonary resistance (R**_L_**) in asthma mice and the levels of CTNNAL1 mRNA in the 8-day time course after the OVA challenge. It is conceivable that CTNNAL1 contributes to BEC constitutive adhesion. In vitro experiments showed that the rate of repair and proliferation of HBEC was slowed down after HBEC was treated with CTNNAL1 ASO, and CTNNAL1 expression was explicitly increased on the cells in wound edges. Those data indicate that CTNNAL1 might be involved in growth regulation and may be beneficial for the recovery of bronchial epithelium damage [8].

In an attempt to identify the response of CTNNAL1 to acute stress, we observed the CTNNAL1 expression under an ozone stressed condition. As shown in this study, CTNNAL1 mRNA was increased both in lungs and in cultured HBEC with the acute ozone stress.

Taken together, our results suggest that CTNNAL1 is involved in maintaining the integrity of the airway epithelium and down regulation of CTNNAL1 expression might contribute to epithelial dysfunction and asthma development. CTNNAL1 upregulation under acute stress conditions might be a protective response. The next question is the mechanisms regarding the regulation of CTNNAL1 expression in HBECs.

Although several studies have shown the significance of altered CTNNAL1 expression, and the exon-intron organization and boundary sequences flanking 19 exons of the human CTNNAL1 gene have been reported recently [2], the mechanism underlying the regulation of CTNNAL1 expression has not been elucidated yet. In order to explore the mechanisms of transcriptional regulation of the CTNNAL1 gene, we identified some potential DNA-binding proteins that can be recruited to CTNNAL1 promoter region and may play roles in the regulation of the transcription of CTNNAL1 gene in this study. Our current findings may help direct further research on the regulation and function of CTNNAL1.

## Materials and Methods

### Ethics Statement

This study was carried out in strict accordance with the recommendations in the Guide for the Care and Use of Laboratory Animals of the National Institutes of Health. The protocol was approved by the Ethics Committee Institute of Clinical Pharmacology of the Central South University (Permit Number: CTXY-090012). All surgery was performed under sodium pentobarbital anesthesia, and all efforts were made to minimize suffering.

### Materials

LightShift Chemiluminescent EMSA Kit was purchased from Pierce Biotechnology. Synthetic oligonucleotides were synthesized by TaKaRa Inc (Dalian China). Anti-LEF-1, anti-AP-2α, anti-CREB polyclonal antibodies and mouse IgG were purchased from Santa Cruz Biotechnology. All chemicals were purchased from Sigma unless stated otherwise.

### Cells culture

The 16HBE14o^−^cells, a generous gift of Dr. Dieter C Gruenert, University of California San Francisco, were SV_40_-transformed human central airway epithelial cells [9]. Cells were cultured in a mixture medium of DMEM: F12 (1∶1) (Sigma, St. Louis, MO) containing 100 u/ml penicillin, 100 u/ml streptomycin, and 10% fetal bovine serum (Hangzhou Sijiqing Biotech. Hangzhou, China) and incubated at 37°C in 5% CO_2_.

### Animal and cell model of ozone exposure

Balb/c mice were obtained from the experimental animal center, Xiangya School of Medicine, Central South University. All mice were housed under specific pathogen-free conditions and had ad libitum access to food and water. The mice housed in whole-body exposure chambers were exposed to ozone concentrations of 2 ppm for 30 min/day for 0,1,2,4,8 days respectively (n = 6/group), while they were awake and breathing spontaneously in the chamber. Cells were exposed to 2 ppm ozone for 30 min under culture conditions using the same equipment as the mice.

### In situ hybridization (ISH) detection for CTNNAL1 mRNA in cultured HBEC and lung tissue

The following probes labeled 5-end with digoxin (purchased from TaKaRa Biotechnology Company) were used for ISH to localize mouse CTNNAL1 mRNA: 5′-ATCACCACACTTATAAACCATAAAGATAATACCAA-3′, 5′-GCATACATGCAGAGAGACATTTCAGGTGACTGGC-3′, 5′-ATGTCCAGGATGGCCTATTCTCTGTATTTATTTAC-3′ or human CTNNAL1 mRNA:5′-ACCTTCCGGAGAAGCCCCAGGAAGACATTA-3′, 5′-ATGGAAGGGGTCTGAGAAGAGATAAGGGCG-3′, 5′-GATCAAAACTCGCTCGGTGGAGCAGACGCT-3′. Briefly, the lung tissues were fixed in 4% paraformaldehyde, embeded in paraffin, cut into 6 mm paraffin sections and placed on slides, the coverglasses with HBEC on them were fixed in 4% paraformaldehyde. They were incubated with 30% H_2_O_2_ and 5% pepsin in turn and then hybridized at 42°C overnight with labeled probes (2.0 g/ml). After washing, the slides and coverglasses were sequentially incubated with blocking buffer, biotin labeled rabbit-anti-digoxin antibody, streptavidin biotin peroxidase complex (sABC) and biotin labeled peroxidase, followed by rinsing with PBS after every step. The peroxidant activity was visualized by the 3, 3-diaminobenzidine tetrahydrochloride (DAB). The coverglasses was incubated with either normal mouse serum instead of the mouse-anti-digoxin antibody or in absence of the labeled probe for the negative control.

### Ozone stress in vitro and flow cytometry

16HBE14O-cells cells was stressed with ozone (1.5 ppm) for 30 min. Then, cells were harvested and washed twice in PBS 12 h after ozone stress. After treated by 1% triton X-100 (Sigma) for 15 min, cells (1×10^5^ cells for each sample) were washed and binded with mouse monoclonal to CTNNAL1 (ab57875, 1∶250 dilution; Abcam, Cambridge, UK). Then, a phycoerythrin (PE) fluorescence second antibody was used to label CTNNAL1 expression by flow cytometry. For all experiments, mean fluorescence intensity (MFI) values were calculated by subtracting primary antibody.

### ASO design

Two ASOs were designed according to mRNA sequences of AP-2*α* (5′-CAGCTGGGGCAACCGTGCCGT-3′ against nucleotides 55–72) and LEF-1 (5′- TCCTCCGGAGAGTTGGGGCAT -3′ against nucleotides 1–21). The sequence of the nonsense oligonucleotide was 5′- GGGGCCCACGGGCAGATCCAT -3′. All ASOs were verified by BLAST and were synthesized (by TaKaRa) as 21-base phosphorothioate oligonucleotides. HBECs were transfected with an ASO and Lipofectin (Invitrogen) mixture for 4 h in serum-free DMEM, and then cultured in normal growth media for 40 h. The effects of the ASOs were measured by real-time PCR after ozone exposure for 30 min and further culture for 4 h.

### Real time PCR measurement

RNA was extracted from mouse lung tissues and cultured HBEC and reverse transcription was performed by *AMV* reverse transcriptase (QIAGEN, Gemany). PCR was then carried out using ShineSybr® Real Time qPCR Kits (Shinegene, China). ShineSybr is a 2× convenient premix reagent, specially designed for real time PCR by using sybr green I. This product combines the high performance of Taq, which is an enzyme for hot start PCR utilizing Taq antibody, with a newly developed buffer which provides superior specificity, increased amplification efficiency and high aptitude for high-speed real time PCR. The primers were synthesized as Table 1. Briefly, 2 µl of the reverse-transcripts was added to a 25 µl PCR mixture for 40 cycles. Each cycle included 93°C for 30 s, 60°C for 30 s and 72°C for 30 s. Normalization of mRNA expression data for sample-to-sample variability in RNA input, RNA quality, and reverse transcription efficiency was achieved by comparing the copy numbers of target mRNAs with that of mouse or human actin or mouse GAPDH for each run.

**Table 1:** Oligonucleotide primers used for real-time PCR analysis.

	Sequence of primers
hCTNNAL1F	5′-GGAGTTTGCACATCTGAGTGGA-3′
hCTNNAL1R	5′-CCAATGCCACTTTCATACGG-3′
hLEF-1F	5′-CACCCATCCCGAGAACATCA-3′
hLEF-1R	5′-GACATGCCTTGTTTGGAGTTGA-3′
hAP-2αF	5′-GTGTCCCTGTCCAAGTCCAAC-3′
hAP-2αR	5′-GACACTCGGGTGGTGAGAGC-3′
h actin F	5′-TGACGTGGACATCCGCAAAG-3′
h actin R	5′-CTGGAAGGTGGACAGCGAGG-3
mCTNNAL1F	5′- TCTTCGGGAGAATGTTTGCTT-3′
mCTNNAL1R	5′- TGTGCTCGTGGCTGGTGTAG-3′
mGAPDH F	5′- TGTGTCCGTCGTGGATCTGA-3′
mGAPDH R	5′- CCTGCTTCACCACCTTCTTGA-3′

### Nuclear protein extraction and electrophoretic mobility shift assay (EMSA)

Nuclear extracts of HBEC were prepared as previously described [10]. Nuclear extract protein concentrations were determined using the Bradford protein assay (Bio-Rad Laboratories, Inc., Hercules, CA). Gel shift assays were performed as described previously [10].

The oligonucleotide probes used in EMSA were designed according to a computer-based search with the software Promoter Scan (PROSCAN Version 1.7 suite of programs developed by Dr. Dan Prestridge) and Transfac [11]encompassing putative binding sites in the *CTNNAL1* promoter.

EMSA was performed using LightShift Chemiluminescent EMSA Kit according to the manufacture's protocol. Nuclear extracts (10 µg protein) were incubated with 1 µg poly[d(I-C)], the binding buffer attached to the kit, and biotin end-labeled oligonucleotide probe for 15 min at room temperature. Bound DNA complexes were separated on a 5% nondenaturing polyacrylamide gel electrophoresis and transferred to a positive nylon membrane. The nylon membranes were UV cross-linked, probed with streptavidin-HRP conjugate and incubated with the chemiluminescent substrate for chemiluminescent detection. For competition experiments, unlabelled competitor oligonucleotides were pre-incubated at 100-fold excess with the labeled probe. The nuclear factors in the retarded bands were verified using the assay of mutated probes binding with extracts and antibody supershift analysis.

### Chromatin immunoprecipitation (ChIP)

To crosslink proteins to DNA, formaldehyde (final concentration 1%) was added to the culture medium and incubated for 10 min at room temperature. Then, a final concentration of 0.125 M glycine was added to stop fixation and cells were scraped and collected. Cells were lysed with SDS lysis buffer (1% SDS, 10 mM EDTA, and 50 mM Tris, pH 8.1) containing protease inhibitors. Aliquots of cell lysates were sonicated to shear DNA into 0.2–1.0 kb-fragments and cellular debris was removed. Chromatin aliquots were precleared with 10 µg of mouse IgG bound to Protein A-Sepharose (GE Healthcare). Samples were then incubated with 1 µg of specific antibodies overnight at 4°C with rotation. Immunocomplexes were mixed with Protein A-Sepharose followed by incubation for 1 h at 4°C with rotation. Beads were collected by brief centrifugation and the immunocomplexes were eluted by freshly prepared elution buffer (100 mM NaHCO3, 1% SDS). Chromatin was then de-crosslinked for 5 h at 65°C. After treated with proteinase K, DNA was phenol/chloroform-extracted, and ethanol-precipitated. An aliquot (2 µl) of each sample was subjected to PCR analysis [12].

### Reporter gene construct and reporter gene assay

The putative promoter of the human CTNNAL1 gene was PCR amplified (−569∼−143 from ATG) and cloned into the Kpn I and Bgl II site of the pGL3-basic luciferase reporter vector (Promega; Madison, WI). This reporter was designated as wild-type CTNNAL1-Luc (Wt- CTNNAL1-Luc) in this study. Nucleotide identity and direction of the insert were verified by sequencing of both strands. Various types of mutations were introduced into Wt- CTNNAL1-Luc using genomic DNA synthesis methods.

Transcription Element Search System (TESS) software was used to analyze all possible binding sites of selected transcription factor on the positive and negative chain of CTNNAL1promoter, and to ensure none of the site-directed mutagenesis of transcription factor binding sites would surplus create new binding sites of other transcription factors.

Cells were seeded onto 24-well culture plates the day before transfection. On the day of transfection, each reporter vector (0.6 µg/well) was transfected into the cells using Lipofectamine 2000 reagent (Invitrogen) according to the manufacture's protocol. For standardization, the phRL-TK vector (Promega) (0.1 µg/well) was also transfected into the cells. Six hours after transfection, the medium was replaced and ozone treatment was added. Twenty-four hours after the addition of the test agents, cells were harvested by Passive Lysis Buffer (Promega), and reporter gene assay was performed with Varioskan Flash multitechnology microplate reader (Thermo Scientific) using Dual-luciferase assay system (Promega). The results represent the average of three independent transfection assays normalized to Renilla reniformis activity.

### Statistical analyses

Data are presented as the mean ± SEM from at least three independent experiments. Statistical difference between two groups was determined by t -test. Multiple comparisons were performed by one-way ANOVA or repeated measures ANOVA together with post-hoc pairwise comparisons. *P*<0.05 was considered statistically significant.

## Results

### The expression of CTNNAL1 in lungs and cultured HBEC

**Figure 1:**
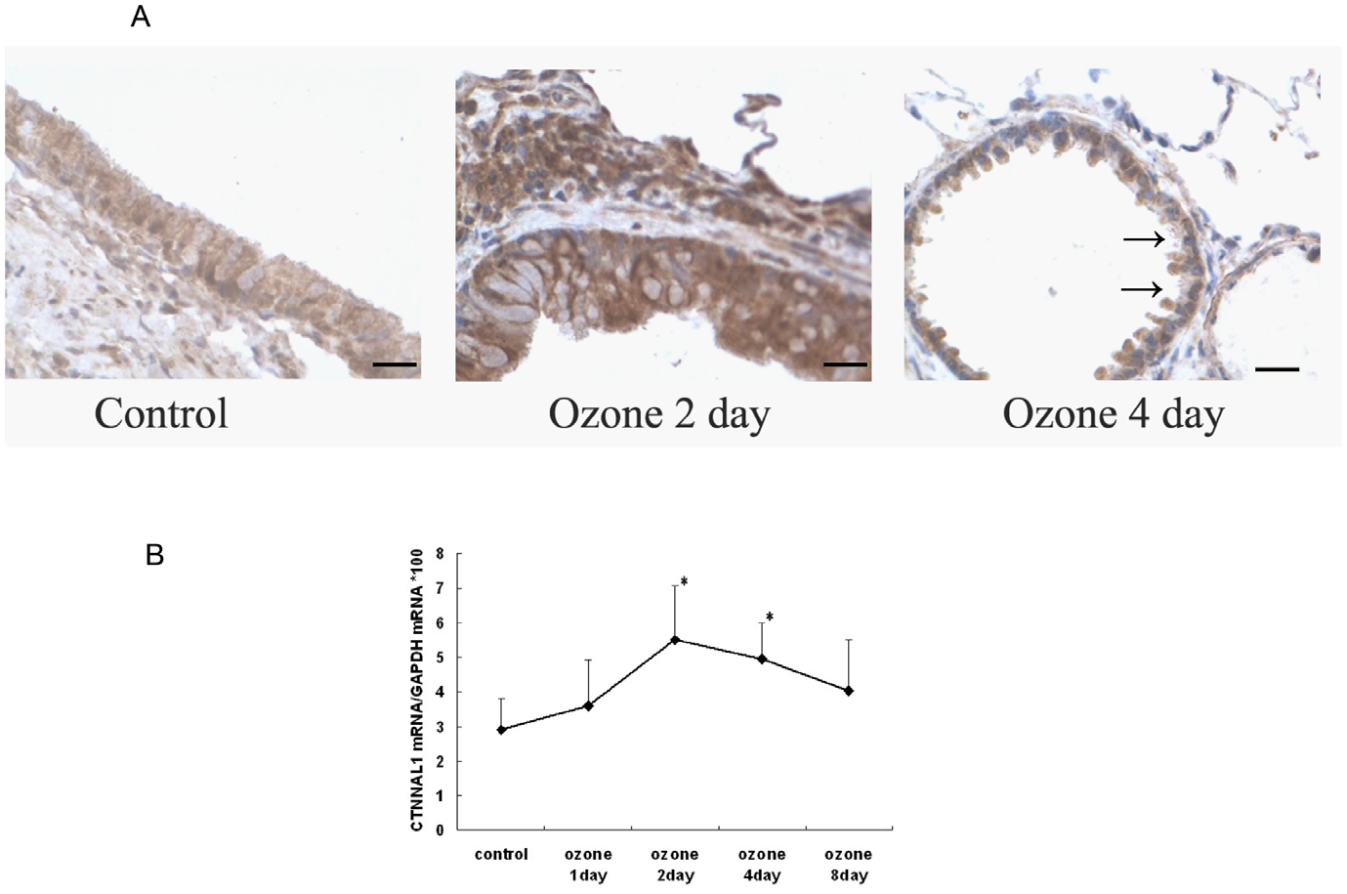
Expression of CTNNAL1 mRNA in ozone stressed mouse lung. (A) It was shown by in situ hybridization that the structure of lung tissues was damaged after being stressed with ozone. Goblet cell hyperplasia and bronchial epithelial denudation were seen in the 2^nd^ and 4^th^ day of ozone stress. The expression of CTNNAL1 mRNA was increased with the ozone stress (×200, bar = 100 µm). Arrows indicate airway epithelium damage. (B) Real time PCR showed the time course of CTNNAL1 expression in mice lung tissue. The result showed that the expression of CTNNAL1 mRNA was increased with the ozone stress, peaked on the 2nd day, and then decreased. (n = 5, *P<0.05).

**Figure 2:**
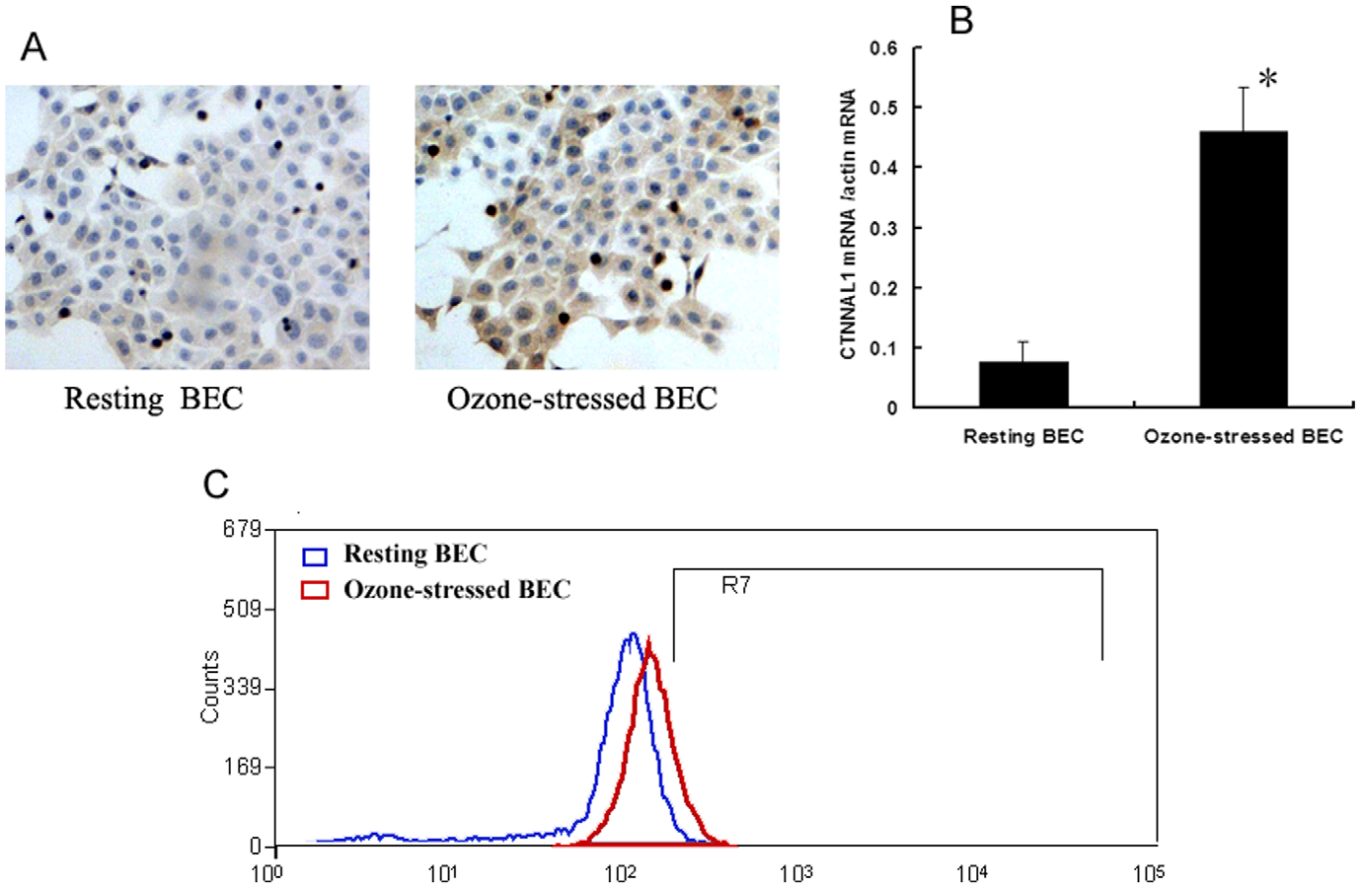
The expression of CTNNAL1 on cultured human BEC was increased significantly after ozone stress. (A) CTNNAL1mRNA expression on BEC assayed by in situ hybridization (DAB staining, ×200). (B) CTNNAL1mRNA expression on BEC assayed by real time PCR (n = 4, *P<0.05). (C) CTNNAL1 protein expression on BEC assayed by flow cytometry detection.

Balb/c mice undergoing 0–8 day's intermittent ozone stress (2 ppm ozone for 30 min/day in whole-body exposure chambers) showed an up-regulation of CTNNAL1 mRNA expression. It was shown that the structure of lung tissues was damaged after being stressed with ozone. Goblet cell hyperplasia and bronchial epithelial denudation were seen in the 2nd and 4th day of ozone stress. In situ hybridization and real-time PCR showed that CTNNAL1 mRNA was increased with the ozone stress, peaked on the second day, and then decreased slightly (Figure 1). In cultured HBEC, after an acute 30 min ozone stress, the expression of CTNNAL1 mRNA was explicitly increased (Figure 2A, B). We also detect CTNNAL1 protein by flow cytometry. The results showed that expression of CTNNAL1 protein was increased after ozone stress (mean fluorescence intensity values 2.18%±0.39% vs 3.58%±0.91%, P<0.05, n = 3, Figure 2C). These results revealed that the expression of CTNNAL1 can respond to ozone stress.

### Identification of transcription factors binding to the human CTNNAL1 promoter region

**Figure 3:**
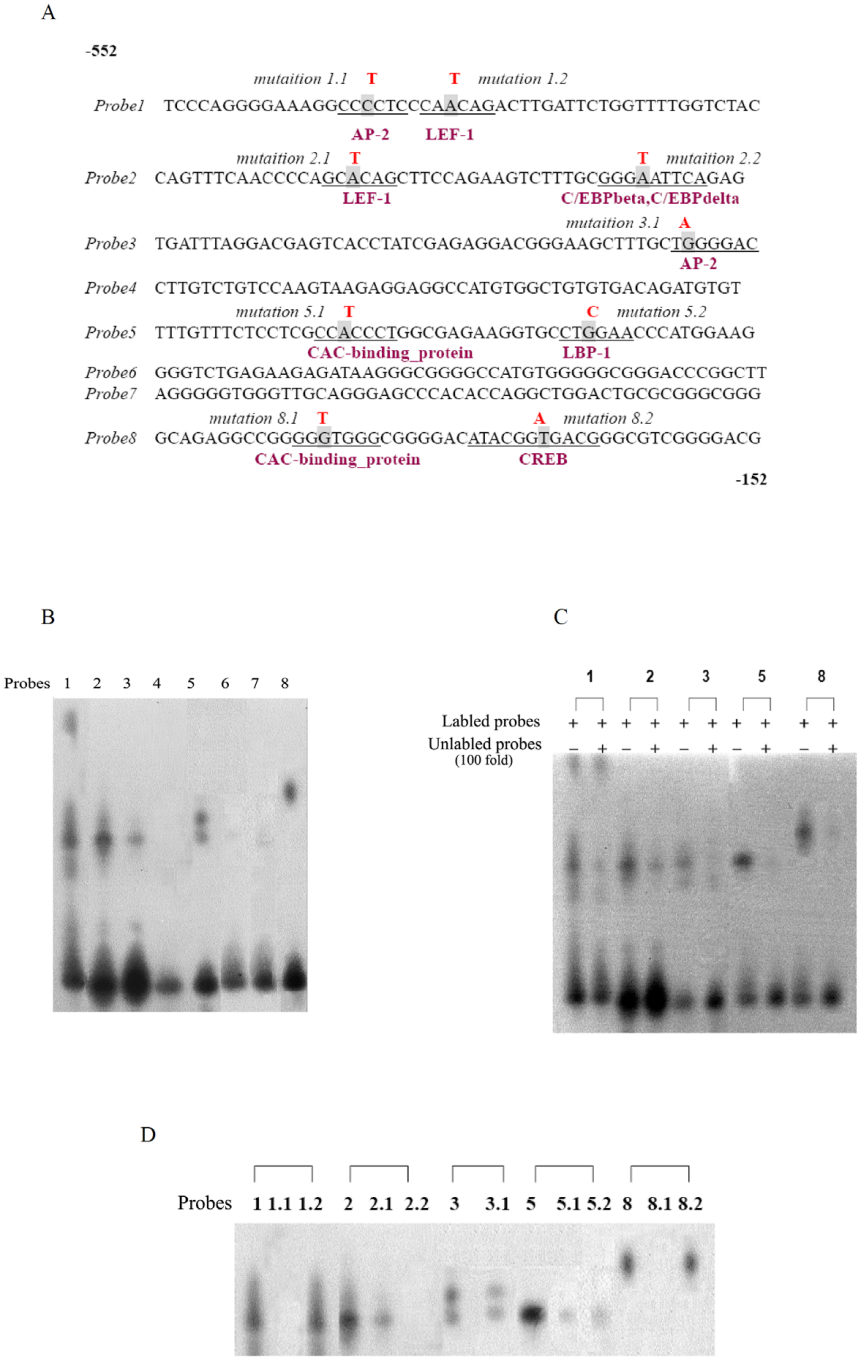
Screen of transcription factors binding to the human CTNNAL1 promoter region. (A) Depicted is the 400 bp DNA sequence of the human CTNNAL1 promoter (−552∼−152 from ATG) which was divided equally to probes 1–8 used for EMSA. Underlines represent possible nuclear-factor-binding sites in corresponding probes. The shaded residues represent the mutated sites in different mutated probes. (B) DNA-protein complexes binding to the human CTNNAL1 promoter were demonstrated in probes 1, 2, 3, 5, and 8. (C) EMSA competiton test of probe 1, 2, 3, 5, and 8. Competition with unlabeled primer significantly reduced the entire five probe's binding intensity. (D) The nuclear factors in the retarded bands were verified using the assay of mutated probes binding with extracts. 9 mutations according to putative binding sites search by TESS were designed as shown in Figure 3A. The valid mutations (specific binding bands was be competed out by unlabelled and 100-fold excess mutation probes) were 1.2, 2.1 (representing the LEF-1 site), 3.1 (representing the AP-2 site) and 8.2 (representing the CREB site).

Analysis with Promoter Scan (PROSCAN Version 1.7 suite of programs developed by Dr. Dan Prestridge) and Transfac [11] on CTNNAL1 gene 5′ non-coding region suggesting that the host range of promoter is −552∼−152 from ATG. In an attempt to identify transcription factors that interact with this region, 8 synthetic DNA probes, designated probe 1–8 (50 bp each, covered the full length of the promoter sequence), were subjected to gel retardation assays (Figure 3A).

The binding reaction of probes with transcriptional factors were observed using EMSA. It was shown that probe 1, 2, 3, 5, and 8 had significant retarded bands (Figure 3B). Competition with 100-fold excess of unlabelled probes revealed specifically DNA–protein binding of these 5 probes (Figure 3C).

The above gel retardation analyses suggest that multiple proteins bind to separate sites on the promoter. Therefore we analyzed further the nature of protein binding to these genomic DNA sequences represented in probes 1, 2, 3, 5, and 8 by introducing specific mutations followed by EMSA. was used to analyze the putative nuclear transcription factor binding sites of each probe [13].

A database search by Transcription Element Search System (TESS) software of the promoter region revealed the presence of several putative binding sites for transcription factors, including AP1and LEF-1 on probe1; LEF-1 and C/EBPbeta on probe2; AP-2 on probe3, CAC-binding_protein and LBP-1on probe5; CAC-binding_protein and CREB on probe8, as shown in Figure 3A. Mutation probes were designed accordingly (Figure 3A) and these mutant probes were unlabelled and 100-fold excess used to competent with the corresponding normal labeled probe. If the mutation is valid, specific binding bands would not be competed out; retarded bands can still be seen. If the mutation is invalid, then the competition can be normal, with the loss of specific bands. Results showed that the valid mutations were 1.2, 2.1 (representing the LEF-1 site), 3.1 (representing the AP-2 site) and 8.2 (representing the CREB site).

**Figure 4:**
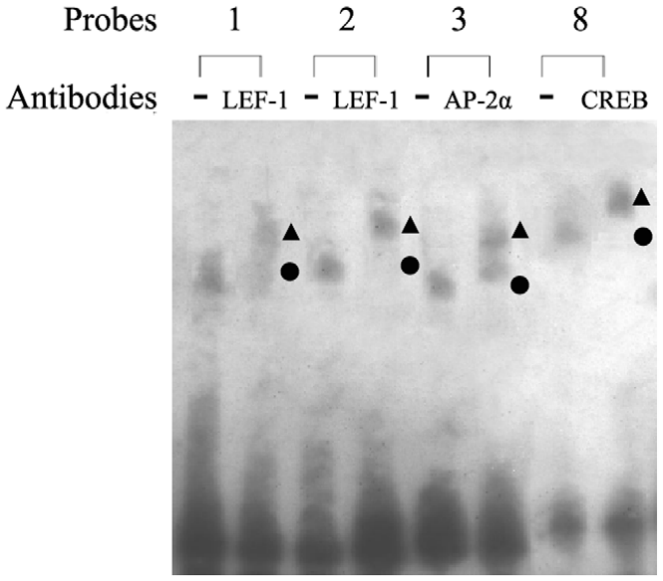
Supershift analyses were performed using nuclear proteins from HBEC that were incubated with specific antibodies against LEF-1, AP-2α, and CREB. The supershifted complex detected with each antibody was indicated by “▴”. Note that each of the LEF-1, AP-2α, and CREB antibodies caused a marked decrease in the intensity of the complex (marked by “•”) formed by the interaction of antibody and nuclear extract.

In super shift experiments with specific antibodies against those transcription factors identified in EMSA and mutational analysis, the three DNA-binding proteins were characterized independently as LEF-1, AP-2α and CREB, as demonstrated by super shifted specific DNA–protein complexes (Figure 4).

**Figure 5:**
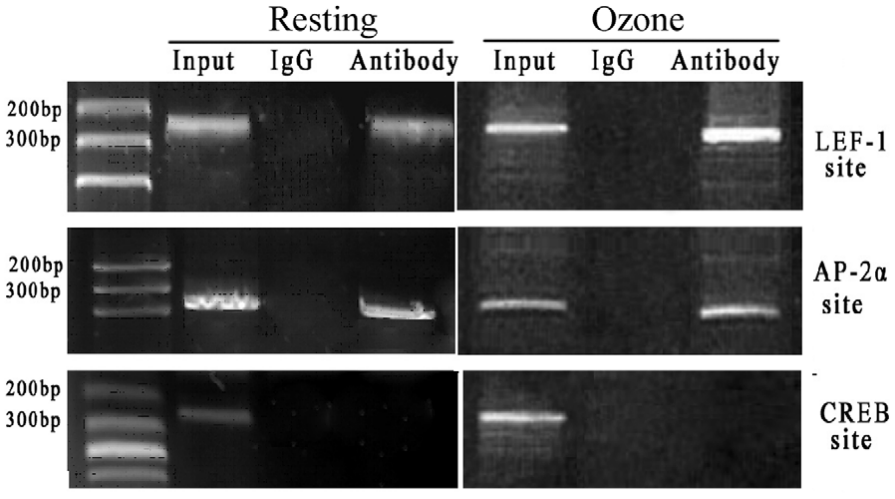
ChIP of three nuclear factors bound to the human CTNNAL1 gene. Cross-linked chromatin prepared from resting BEC or ozone-stressed BEC was immunoprecipitated with three kinds of specific antibodies respectively, or nonimmune IgG as indicated.

Next, we investigated whether these three nuclear factors could, “in vivo”, physically bind the human CTNNAL1 promoter. The binding of transcription factors to the CTNNAL1 promoter with or without ozone stress was verified by chromatin immunoprecipitation (ChIP) assays (Figure 5). HBECs were exposed to formaldehyde to cross-link proteins and DNA, and were sonicated to fragment the chromatin. Specific antibody against LEF-1, AP-2α, CREB or normal rabbit IgG were used to immunoprecipitate the protein-DNA complexes. After immunoprecipitation, DNA was extracted from the beads and used as a template to generate specific PCR products. The presence of the promoter specific DNA region before immunoprecipitation was confirmed by PCR (input). PCR product was observed in LEF-1 and AP-2α immunoprecipitation groups but not observed in CREB immunoprecipitation group, which indicating an interaction between LEF-1 or AP-2α and CTNNAL1.

### Transcriptional regulation of human CTNNAL1 in BEC

To further confirm whether LEF-1 and AP-2α can activate transcription of CTNNAL1, CTNNAL1 promoter gene was inserted into luciferase reporter plasmid pGL3-Basic to construct the human CTNNAL1 promoter–luciferase reporter pGL3/CTNNAL1/luc.

TESS software was used to analyze all possible binding sites of LEF-1 and AP-2α in positive and negative chain of CTNNAL1 promoter and found that LEF-1 had 5 possible binding sites, while the AP-2α had 7 possible binding sites (Table 2). These multi-copy loci indicate both LEF-1 and AP-2α might have important roles in the regulation of transcription of CTNNAL1. We mutated all the possible binding sites of LEF-1 or AP-2α on the luciferase reporter plasmid containing the CTNNAL1 promoter.

**Table 2:** Site-directed mutation position of two nuclear factors.

	Sequences
LEF-1	5′- CCCCTC CCA***T*** (−529 bp A→T) CAGACTTGAT CAGC ***T*** (−486 A→T)CAGCTTCC
	3′-GGGGAG GGT***A*** (−529 bp T→A) GTCTGAACTAGTCG ***A*** (−486 T→A)GTCGAAGG
	AGAAGTCTT ***A*** (−468T→A)GCGGAGGACGGGAAGCTT ***A*** *(−412 T→A)* GCTGGG
	TCTTCAGAA ***T*** (−468A→T)CGCCTCC T GCCCTTCGAA ***T*** (−412 A→T) CGACCC
	GACGCAGGCCATGTGGCTG ***A*** (−366 T→A)GTACAGATGTGTTT-3′
	CTGCGTCCGG TACACCGAC ***T*** (−366 A→T) CATGTCTACACAAA-5
AP-2α	5′-TCC ***T*** (−549 C→T)AGGGGAAAGGCC ***T*** (−536 C→T)CTCCCAAGAAGCTTTGCT
	3′-AGG ***A*** (−549 G→A)TCCCCTTTCCGG ***A*** (−536 G→A)GAGGGTTCTTCGAAACGA
	***A***(−408 G→A)GGGACGCTTGTAAGAGATAAGGGC ***T*** *(−280 G→T)* GGGCC ATG
	***T*** (−408 C→T)CCCTGCGAACA TT CTCT ATTCCCG ***A*** (−280 C→A) CCCGGTAC
	CTTAGGGGGTG ***T*** *(−244 G→T)* GGTTGCAGGGAGGCCGG ***T*** (−191 G→T)GGT G ***T***
	GAATCCCCCAC ***A*** (−244 C→A)CCAACGTCCC TCCGGCC ***A*** (−191 C→A)CCAC ***A***
	(−186 G→T)GCGGGGACA-3′
	(−186 C→A)CGCCCCTGT-5′

**Figure 6:**
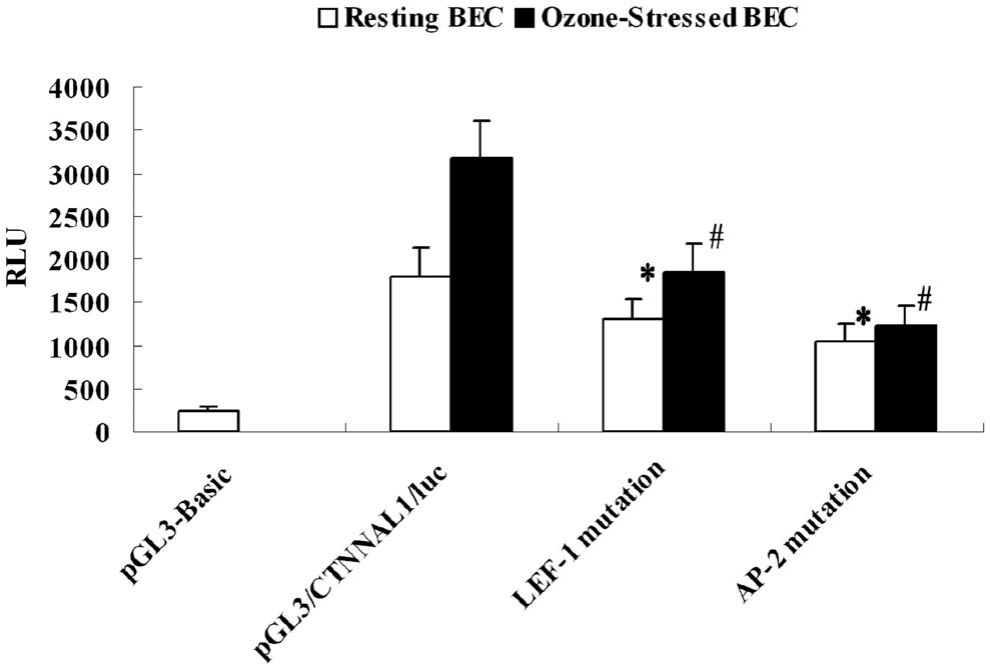
Effects of site-directed mutation in transcriptional factors LEF-1 and AP-2α on transcriptional activation of the human CTNNAL1 luciferase reporter. Shown is transcriptional activation of the human CTNNAL1 luciferase reporter in BEC. Data represent the means of relative luciferase activities (normalized to Renilla reniformis) from at least 5 independent transient transfection experiments each performed in triplicate. (^*^P<0.05 versus pGL3/CTNNAL1 under resting condition; ^#^P<0.05 versus pGL3/CTNNAL1 under ozone-stressed condition).

The above plasmids were transiently transfected into human bronchial epithelial cells. cells were then collected 48 h after transfection to determine luciferase chemiluminescence signal strength (RLU, relative chemiluminescence intensity). As shown in Figure 6, transient transfection with the luciferase reporter 426 bp (−569∼−143 from ATG) luc-construct resulted in an increase in luciferase activity relative to the empty promoter less pGL3-basic vector, demonstrating that this DNA fragment contains significant promoter activity in HBEC (5–10 fold increase). Results from similar transfection experiment under ozone-stressed conditions demonstrated a boost activation of the human CTNNAL1 promoter. After LEF-1 binding site mutation, RLU in normal cells and ozone stressed group decreased 28.3% and 42.1% respectively; after AP-2α binding site mutation, RLU in normal cells and ozone stressed group were reduced 41.7% and 61.1% respectively (Figure 6). These results suggest that, LEF-1 and AP-2α binding sites were required for the maintenance of CTNNAL1 promoter activity, this effect is more pronounced under ozone stress.

### Regulation of LEF-1 and AP-2α on CTNNAL1 expression in ozone stressed HBEC

**Figure 7:**
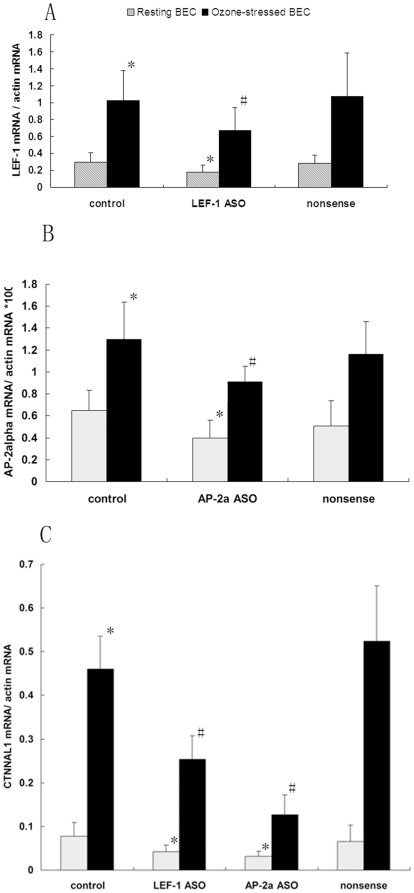
Inhibition of transcription factors and human CTNNAL1 expression subsequent to ASO treatment. (A) (B) The mRNA expression levels of LEF-1 and AP-2αin ozone-stressed HBEC were assessed by real-time PCR with and without ASO treatment. (C) Human CTNNAL1 mRNA expression was significantly induced by ozone exposure. The inducible expression was substantially reduced after ASO treatment specifically targeting LEF-1 and AP-2α. Results are means±S.D. for four experiments. *P<0.05 compared with resting BEC group; #P<0.05 compared with ozone-stressed BEC group without ASO treatment.

To investigate whether LEF-1 and AP-2α were involved in the CTNNAL1 mRNA up-regulation in ozone stressed HBEC, ASOs targeting these two transcription factors were designed, and treatment efficiency was assessed by real-time PCR. The results showed that both ASOs caused substantial reduction in mRNA expression levels in normal and ozone-stressed HBEC, while the nonsense oligonucleotide had no effect (Figure 7A, B). CTNNAL1 mRNA expression was significantly up-regulated in ozone-stressed BEC (Figure 7C), whereas pre-treatment with ASOs targeting LEF-1and AP-2α yielded significant reduction of ozone-stress-induced CTNNAL1 expression. Human CTNNAL1expression under resting conditions could also affected by pre-treatment with ASOs, with the lower degree than that of ozone-stressed HBEC, (Figure 7C).

### Time course of LEF-1, AP-2α and CTNNAL1 expression in ozone stressed HBEC

To further delineate the potential role of LEF-1 and AP-2α in CTNNAL1 regulation in HBEC, a 16 h time course of LEF-1 and AP-2α DNA-binding activity and CTNNAL1 expression was examined after 30 min of ozone stress using EMSA (with probes 1 and 3 respectively) and real-time PCR respectively. The results showed that the activation of LEF-1 occurred within 30 min and peaked at 1 h after ozone exposure. The activation of AP-2α occurred within 1 h of ozone exposure, peaked at 30 min and decreased at 4 h.

**Figure 8:**
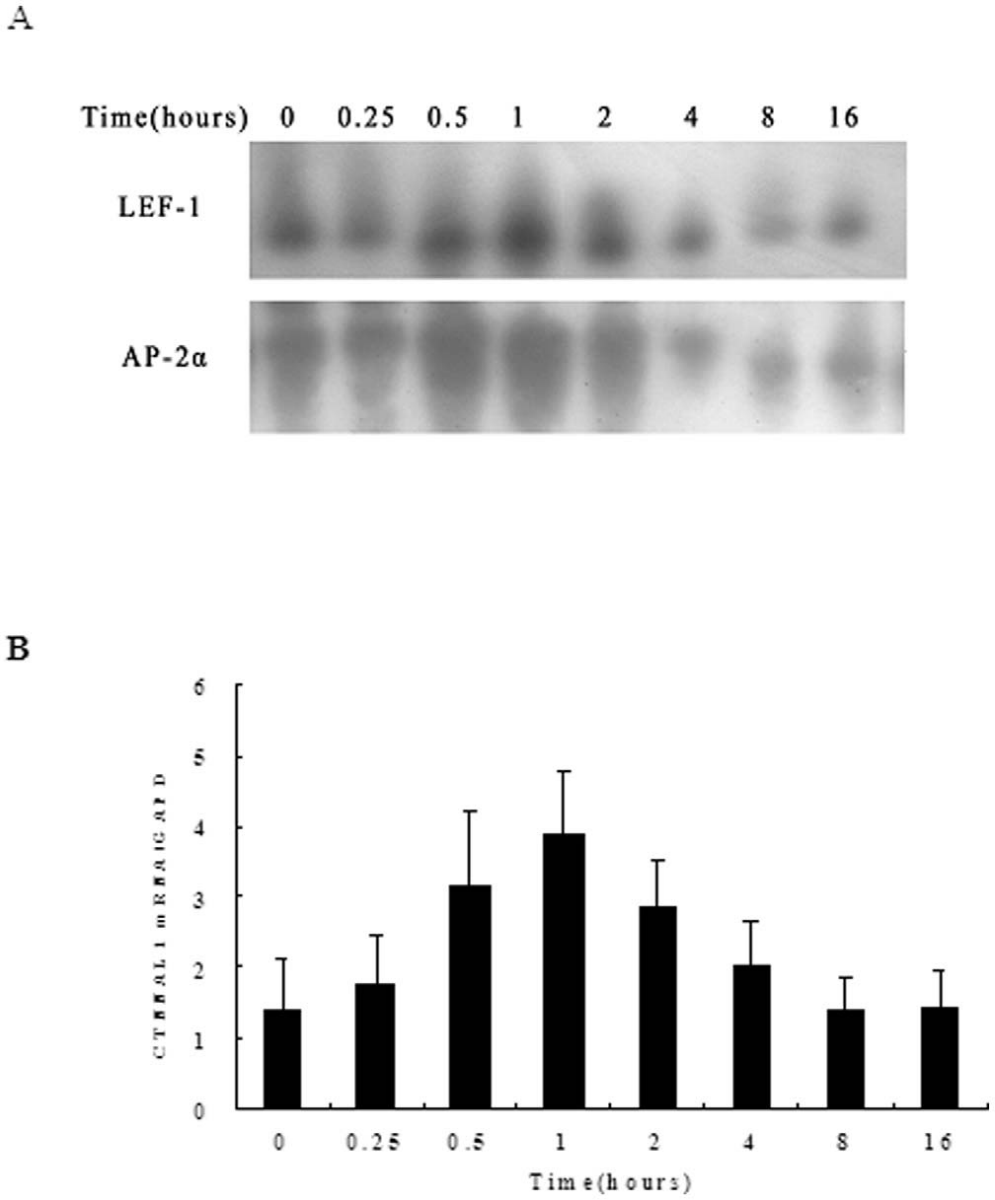
Time course of human CTNNAL1 mRNA expression and DNA-protein binding activities of AP-2α and LEF-1. (A) The time course of AP-2α and LEF-1 DNA binding to CTNNAL1 promoter sequence under ozone stress was determined by EMSA. Expression of human CTNNAL1 mRNA was assayed by real-time PCR. Data represent means ± SD of 4 independent experiments.

CTNNAL1 mRNA expression as measured by real-time PCR followed the pattern of LEF-1 and AP-2α activation. A gradual increase of human CTNNAL1 expression occurred until it reached the peak at 1 h, and then decreased (Figure 8 A, B).

## Discussion

Transcription factors tightly regulate gene expression in response to intra- and extracellular stimuli, and they often play a central role in determining cell fate by controlling the fundamental mechanism of gene transcription. In order to elucidate the mechanism responsible for CTNNAL1 gene expression, we designed a protocol in the present study to identify the putative DNA-binding proteins regulating the transcription of CTNNAL1 gene. First, we speculated the putative promoter using a computer-based search with the software Promoter Scan (PROSCAN Version 1.7) and Transfac, by which a 400 bp DNA fragment (−552∼−152 from ATG) was determined to be the promoter region. Next, 8 oligonucleotide probes, 50 bp each, were designed, covering the overall promoter region. The spectrum of potential nuclear factors involved in expression of CTNNAL1 was screened using EMSA. On the basis of the assay of mutated probes and antibody supershift, they were verified as LEF-1, AP-2α and CREB. Then ChIP assay was used to verify the in vivo interaction between these transcription factors and CTNNAL1 promoter. Only AP-2α and LEF-1 show the binding on CTNNAL1 promoter. By site-directed mutagenesis of putative transcription-factor-binding sites within pGL3/FR/luc, we observed a reduction in human CTNNAL1 promoter activity of mutants of both AP-2α and LEF-1 sites. The time courses showed that ozone stress can activate the AP-2α and LEF-1 within one hour. Ozone-inducible CTNNAL1 expression correlated with AP-2α and LEF-1 binding activity during a-16 hour time course. Furthermore, we investigated if LEF-1 and AP-2α were involved in the mRNA overexpression of the CTNNAL1 gene in ozone stressed HBEC. ASOs targeting the two transcription factors were transfected into the HBEC, and significant reduction of ozone-stress-induced CTNNAL1 expression were observed by pre-treatment with these two ASOs. Our data suggest that a robust transcriptional CTNNAL1 up-regulation occurs during acute ozone-induced stress and is mediated at least in part by ozone-induced recruitment of LEF-1 and AP-2α to the human CTNNAL1 promoter.

Lymphoid Enhancer binding Factor-1 (LEF-1) is a nuclear high mobility group (HMG) protein that mediates gene transcription in response to canonical Wnt/beta-catenin signaling pathway [14], [15]. LEF-1 protein is expressed in a variety of organisms and its expression and functional abnormalities can lead to abnormal development of the organisms. Wnt signaling is involved in virtually every aspect of embryonic development and also controls homeostatic self-renewal in a number of adult tissues. Germline mutations in the Wnt pathway cause several hereditary diseases, and somatic mutations are associated with cancer of the intestine and a variety of other tissues [15]. LEF-1 acts as a central transcription mediator of Wnt signaling, regulating cell cycle and growth-relevant genes like Cyclin D1 and c-myc [16]–[18].

Merdek KD et al reported α-catenin and CTNNAL1 had distinct activities that down regulate β-catenin and Ras signals, respectively, on the cyclin D1 promoter [3]. Our finding that LEF-1, a β-catenin associated nuclear transcription factor, had the effect to regulate CTNNAL1 expression, provided a clue for an interaction between CTNNAL1 and β-catenin signaling pathway. Interestingly, the sub-cellular localization showed expression of α-catenin in the cell membrane, while the CTNNAL1 and β-catenin both in cytoplasm and nucleus [19], which might provide some support for the speculation.

AP-2α is a homodimeric 100 kDa transcription factor with a core recognition element sequence of 5′-GCCNNNGGC-3′ [20], [21]. AP-2α mediates transcriptional activation in response to two signal transduction pathways: the phorbol ester/diacylglycerol-inducible protein kinase C pathway and the cAMP-dependent protein kinase A pathway [22]. Befitting a role of AP-2α in differentiation, stimulatory response elements for this transcription factor have been described in numerous gene promoters [23]. The expression of AP-2*α* is associated with the embryonic differentiation and cancer [24], [25]. Also, AP-2*α* has been shown to regulate neuropeptide receptors [26], [27] and neuropeptide genes [28]. However, the activation and translocation of AP-2*α* remained unclear.

LEF-1 and AP-2α are transcription factors closely linked to cell growth, differentiation and apoptosis. Our data showed that LEF-1 and AP-2α can regulate the expression of CTNNAL1, suggesting CTNNAL1 may play a role in cell growth and wound repair. In the present study, we observed that the ozone-inducible LEF-1 and AP-2*α* activation and the expression of CTNNAL1 mRNA was consistent with their activation, indicating that LEF-1 and AP-2*α* jointly regulate the expression of CTNNAL in response to ozone. However, how the early signals are transmitted to LEF-1 and AP-2*α* and how the early signal molecules assemble with these two facts in response to ozone require further studies.

Overall, these observations encourage our next step of investigation on the presence of these two transcription factor binding site mutation in asthma patients, searching for new molecular evidence of asthma susceptibility.
